# Deciphering Tumor Niches: Lessons From Solid and Hematological Malignancies

**DOI:** 10.3389/fimmu.2021.766275

**Published:** 2021-11-10

**Authors:** Stéphane J.C. Mancini, Karl Balabanian, Isabelle Corre, Julie Gavard, Gwendal Lazennec, Marie-Caroline Le Bousse-Kerdilès, Fawzia Louache, Véronique Maguer-Satta, Nathalie M. Mazure, Fatima Mechta-Grigoriou, Jean-François Peyron, Valérie Trichet, Olivier Herault

**Affiliations:** ^1^ Centre National de la Recherche scientifique (CNRS) GDR3697, Micronit “Microenvironment of Tumor Niches”, Tours, France; ^2^ INSERM UMR1236, Rennes 1 University, Etablissement Français du Sang Bretagne, Rennes, France; ^3^ Cancéropole Grand-Ouest, NET network “Niches and Epigenetics of Tumors”, Nantes, France; ^4^ Saint-Louis Research Institute, University of Paris, EMiLy, INSERM U1160, Paris, France; ^5^ The Organization for Partnerships in Leukemia (OPALE) Carnot Institute, The Organization for Partnerships in Leukemia, Paris, France; ^6^ Center for Research in Cancerology and Immunology Nantes-Angers (CRCINA), Signaling in Oncogenesis Angiogenesis and Permeability (SOAP), INSERM UMR1232, Centre National de la Recherche scientifique (CNRS) ERL600, Université de Nantes, Nantes, France; ^7^ Integrated Center for Oncology, St. Herblain, France; ^8^ Centre National de la Recherche scientifique (CNRS) UMR9005, SYS2DIAG-ALCEDIAG, Montpellier, France; ^9^ INSERM UMRS-MD1197, Paris-Saclay University, Paul-Brousse Hospital, Villejuif, France; ^10^ Cancer Research Center of Lyon (CRCL), CNRS UMR5286, INSERM U1052, Lyon 1 university, Lean Bérard Center, Lyon, France; ^11^ INSERM U1065, C3M, University of Côte d’Azur (UCA), Nice, France; ^12^ Stress and Cancer Laboratory, Institut Curie, INSERM U830, Paris Sciences et Lettres (PSL) Research University, Team Babelized Ligue Nationale Contre le Cancer (LNCC), Paris, France; ^13^ INSERM UMR1238 Phy-Os, Université de Nantes, Nantes, France; ^14^ Centre National de la Recherche scientifique (CNRS) ERL7001 LNOx, EA7501, Tours University, Tours, France; ^15^ Department of Biological Hematology, Tours University Hospital, Tours, France

**Keywords:** microenvironment, cancer-associated fibroblasts (CAFs), mesenchymal stem/stromal cells (MSCs), cytokines and chemokines, energy/oxidative metabolism, mitochondrial transfer, angiogenesis, endothelial plasticity

## Abstract

Knowledge about the hematopoietic niche has evolved considerably in recent years, in particular through *in vitro* analyzes, mouse models and the use of xenografts. Its complexity in the human bone marrow, in particular in a context of hematological malignancy, is more difficult to decipher by these strategies and could benefit from the knowledge acquired on the niches of solid tumors. Indeed, some common features can be suspected, since the bone marrow is a frequent site of solid tumor metastases. Recent research on solid tumors has provided very interesting information on the interactions between tumoral cells and their microenvironment, composed notably of mesenchymal, endothelial and immune cells. This review thus focuses on recent discoveries on tumor niches that could help in understanding hematopoietic niches, with special attention to 4 particular points: i) the heterogeneity of carcinoma/cancer-associated fibroblasts (CAFs) and mesenchymal stem/stromal cells (MSCs), ii) niche cytokines and chemokines, iii) the energy/oxidative metabolism and communication, especially mitochondrial transfer, and iv) the vascular niche through angiogenesis and endothelial plasticity. This review highlights actors and/or pathways of the microenvironment broadly involved in cancer processes. This opens avenues for innovative therapeutic opportunities targeting not only cancer stem cells but also their regulatory tumor niche(s), in order to improve current antitumor therapies.

## Introduction

The bone marrow (BM) is the site where hematopoietic stem cells (HSCs) sustain hematopoiesis after birth and all lifelong in mammals. From early progenitors to committed subsets of myeloid and lymphoid lineages, proliferation and differentiation mechanisms have been extensively studied. They were shown early on, through the use of *in vitro* cultures, to be controlled by cells from the BM microenvironment (1, 2) comprising mesenchymal stem/stromal cells (MSCs), endothelial cells (ECs) and macrophages (3). Since the beginning of the 2000’s, the identity and organization of BM niches supporting hematopoiesis have been extensively studied through the use of reporter mouse models (reviewed in (4, 5)). Furthermore, the heterogeneity of BM MSCs and ECs has recently been approached by single-cell RNA sequencing, which has confirmed the presence of multiple subpopulations within these two cell types (6–10). Beside this heterogeneity of the BM microenvironment, it is now moreover clear that the “one progenitor/one niche” rule does not prevail. Indeed, a single niche can support not only hematopoietic subsets at distinct developmental stages, but also mature immune cells homing back to the BM (5, 10).

Because of the contained location of the BM, knowledge on human hematopoietic niches is more limited even if strong similarities could be observed with mice (10–12). For the same reason, the nature of human leukemic niches and the molecular mechanisms regulated by/within them are still unclear and results strongly rely on *in vitro* cultures or on observations obtained in syngeneic or xenograft mouse models [reviewed in (13, 14)]. Furthermore, the immune microenvironment in tumoral BM is also poorly resolved and despite the tremendous progress that came with the use of immunotherapies, resistance and relapse in acute leukemia still concern many patients. Lessons could be learned from solid tumors for which the easier study of microenvironment (i.e., after surgery) has led to important advances. As in hematopoietic malignancies, a complex crosstalk exists between the tumor and the non-malignant cells in its microenvironment. Interestingly, BM is a haven not only for normal and pathological hematopoietic cells from the periphery, but also for metastatic cells from solid tumors, indicating that at least some properties or components of the tumor microenvironment must be shared between hematopoietic and solid tumors.

Tumor development depends on a multidirectional crosstalk between tumor cells, mesenchymal/endothelial cells and immune cells. The immune landscape and modulation of immune responses exerted by tumor cells, directly or through systemic disruption, have been extensively studied (15–17). In this review, we propose to confront and combine the knowledge gained on stromal/endothelial niches of leukemic and solid tumors by focusing on the BM as a common niche.

## Heterogeneity of CAFs/MSCs

The term “mesenchymal” is widely spread in the literature to designate stromal cells from the microenvironment of many tissues. MSCs are stromal cells able to adhere *in vitro* to plastic and spread on culture plates as spindle-cells or fibroblast-like cells. Specific MSC shapes are associated with differentiation, for instance rounded MSCs during adipogenic differentiation (18). MSCs are characterized by a specific pattern of surface markers. Namely, they express CD105, CD73, CD90 and CD146 in the absence of CD45, CD34, CD14, CD11b, HLA-DR and lymphocyte-lineage markers. MSCs secrete components of the extracellular matrix (*e.g.* collagens, heparan sulphate, elastin, aggrecan) and metalloproteinases as well as a large variety of mitogenic growth factors, cytokines, chemokines and angiogenic factors (19). These cells have also retained the ability to differentiate into osteoblasts, chondroblasts and adipocytes (20). However, inconsistent definitions of MSCs and varying isolation and culture conditions have resulted in highly diverse outcomes and confusing data (21, 22). The mesenchyme does not constitute a lineage but is an embryonic tissue able to give rise to connective tissue, blood vessels and blood cells that can have different embryonic origins. Therefore, there are no common MSCs in adult tissues, reflecting the fact that the nature and properties of the globally termed “MSCs” likely represent different cellular entities (21, 22). Since their initial definition in the early 1990’s (23), the properties of MSCs have been largely explored and debated, even with an attempt at establishing a molecular signature (24). However, a consensus on the definition and use of the term “MSC” is unlikely to be reached. Indeed, with the major improvement of “omic” technological tools in the past years, in particular at the single-cell level, it has become quite obvious that MSCs encompass different subpopulations and states of stromal cells and even fibroblasts. The recent molecular mapping of murine BM niche populations, under homeostatic conditions, by single-cell RNA sequencing, clearly demonstrated a great cellular heterogeneity of BM stromal cells (6–8, 10). This heterogeneity was also identified in human MSCs (from umbilical cord), among which two groups were separated based on differentially expressed genes, including CD73. The first group is characterized by an enriched expression of genes involved in immune response/regulatory activities, muscle cell proliferation and differentiation, stemness and oxidative stress. The second presents a higher expression of genes involved in extracellular matrix production, osteoblast and chondrocyte differentiation, and bone and cartilage growth (25).

**Figure 1:**
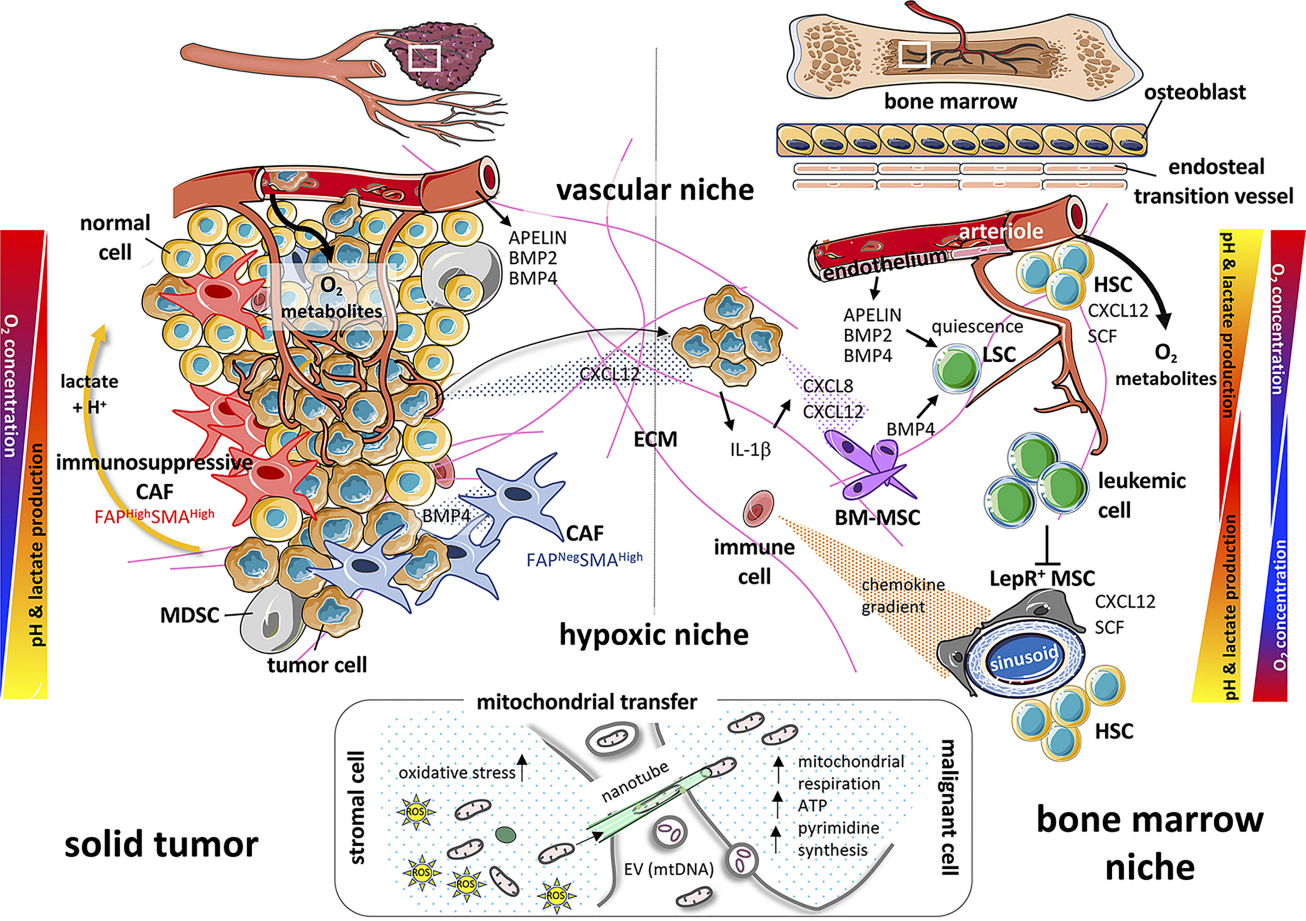
Paralleling the microenvironment and actors of the solid tumor and bone marrow niche. On the left part of the diagram, the solid tumor is governed by cellular components such as healthy cells and tumor cells juxtaposed with immunosuppressive or neutral cancer-associated fibroblasts (CAFs) and myeloid-derived suppressor cells (MDSCs). The different gradients of oxygen, pH and growth factors (BMP2, BMP4), then participate in the tumor cell fate (proliferation, metastasis…). On the right panel, healthy hematopoietic stem cells (HSCs), bone marrow-mesenchymal stem/stromal cells (BM-MSCs), immune cells and tumor cells (attracted from a solid tumor by chemokine gradients such as CXCL12) or leukemia cells, quiescent or not, will be confronted with gradients of oxygen, pH, growth factors (BMP2, BMP4, SCF, APELIN) and cytokines (CXCL8, CXCL12, IL-1β) in a similar way as cells within the solid tumor. All these interrelations and interconnections, controls and feedbacks, will allow the tumor cell to proliferate and spread. Oxygen gradients, on the left, result from diffusion of oxygen from the blood vessels (vascular niche) as tumors grow outward from the local vascular architecture. Vascular niche may also be a source of factors favoring tumor growth (*i.e* Apelin) in solid tumor and in the bone marrow niche. On the right, there is a double gradient between arterioles and sinusoids. The cells will adapt their metabolism along these gradients and create, as a counterpart, a pH gradient due to the release of lactate and H^+^ protons. Bottom part: As crucial powerhouses for cell metabolism and tissue survival, mitochondria will transfer horizontally from stromal cells to cancer and/or immune cells, *via* nanotubes, extracellular vesicles (EVs) or freely, to allow recipient cells to adapt and modify their metabolism (mitochondrial respiration, ATP, pyrimidine synthesis) to meet different stresses (oxidative stress) and energy demands.

In a malignant context, and more particularly in acute myeloid leukemia (AML), the most recent studies report functional abnormalities of human MSCs, which have a significant impact on the aggressiveness of the disease. Among these anomalies, growth deficiency, altered osteogenic differentiation ability and reduced capacity to support hematopoietic cells (26–31) have been described, as well as modifications of the secretome (28, 32), which induce *in vivo* shaping of the stromal niche by leukemic cells (33). Moreover, single cell analyses of murine BM stromal cells recently revealed that leukemia remodels the BM stroma to the disadvantage of normal hematopoietic cells. This notably involves a blockade of the osteoblastic development, as well as of the pathway of bone morphogenetic proteins (BMPs), including *Bmp4.* It also induces a decreased expression of *Cxcl12* and *Kitl* by leptin receptor expressing osteoprogenitors that regulate HSCs (7) (**Figure 1**).

All solid tumors contain non-tumor stromal supporting cells which are also called tumor-associated stromal cells. In carcinomas, they are well-known as cancer-associated fibroblasts (CAFs). Heterogeneity of tumor-associated stromal cells between tumors and, more recently, within a single tumor, has been disclosed, essentially through flow-cytometry, sorting and single-cell RNA sequencing. Their role in tumor progression is still explored with the use of mouse models. In line with such analyzes, four CAF subsets have been identified in breast and ovarian cancers by combining the study of distinct CAF markers, including the fibroblast activation protein (FAP), smooth-muscle α-actin (SMA) and integrin β1 (CD29) (34–37). Two subsets have also been detected in healthy tissues, reminiscent of normal fibroblasts, while two myofibroblastic subsets (FAP^High^ SMA^High^ CD29^Med-High^ and FAP^Neg^ SMA^High^ CD29^High^) appear to be restricted to tumors (**Figure 1**). These CAF subsets are respectively characterized by their secretion and organization of extracellular matrix (ECM) components, in particular types 1, 3 and 6 collagen, and by a perivascular contractile gene signature (34, 35, 37). Importantly, the association of the FAP^High^ SMA^High^ CAF subset with poor outcome has been validated by different laboratories in mouse models, as well as in some types of human carcinoma (38–42), highlighting its relevance in distinct species and cancer types.

Consistent with their accumulation in aggressive carcinomas, FAP^High^ SMA^Med-High^ CAFs favor metastatic spread in breast and ovarian cancers by directly interacting with cancer cells, and the highly contractile FAP^Low^ SMA^High^ CAFs promote cancer cell invasion in 3-dimensions by remodeling the surrounding ECM (34–37, 43–45). Osteosarcoma-associated stromal cells have been characterized by MSC markers and SMA expression, like their healthy counterparts, but with a higher osteoblastic potential and an increase in lung metastases in mouse models (46). Mimicking the acidity of tumor microenvironment has been shown to lead osteosarcoma-associated MSCs to acquire an inflammatory phenotype, with an increased secretion of IL-6 and CXCL8. Such conditions also promoted the stemness of osteosarcoma cells (47). By contrast, healthy MSCs did not modify the quiescent state of osteosarcoma cells (48). Osteosarcoma-associated MSCs were moreover shown to promote not only the invasiveness of osteosarcoma cells, but also angiogenesis through the activation, proliferation and/or differentiation of ECs (49).

Myofibroblastic CAFs (SMA^+^) secrete type-I collagen that can modulate immune cells. In a mouse model of pancreatic adenocarcinoma, it was recently demonstrated that the reduction of total type-I collagen secreted by CAFs accelerated the emergence of carcinoma. This was shown to be due to an upregulation of the chemokine CXCL5 (see next section) in cancer cells, leading to the recruitment of myeloid-derived suppressor cells (MDSCs) and impairment of CD8^+^ cytotoxic T-cells (50). Conversely, the secretion of matrix metalloproteinases (MMPs) can favor tumor cell mobility across collagen fibers and inhibit immune cell activity. Melanoma-associated fibroblasts have thus been described as negative immuno-modulators, through the secretion of MMPs decreasing tumor cell lysis by natural killer (NK) cells (51). The ECM composition can be modified by the protease activity of FAP, which is expressed by tumor and stromal cells in many human carcinomas and sarcomas (43, 52, 53). Consistent with these observations, FAP^High^ SMA^+^ CAFs have been identified in aggressive carcinomas to exhibit immunosuppressive activities (43, 44). Indeed, FAP^High^ CAFs are associated with immunosuppression and resistance to immunotherapies in mouse models (37, 54, 55). Interestingly, the FAP^High^ SMA^+^ CAF subset promotes immunosuppression by increasing the infiltration of regulatory T lymphocytes (Treg) in human cancers (34, 35). Within the FAP^High^ CAF subpopulation, two distinct subsets exhibiting either an ECM-producing myofibroblastic phenotype (myCAF) or an inflammatory profile (iCAF) were recently identified in different types of cancers (56–60). Importantly, recent single cell sequencing of FAP^High^ CAFs from breast cancer cells allowed identifying eight different FAP^High^ cellular clusters (59). Three of them were further shown to be specifically associated with resistance to immunotherapy in metastatic melanoma and in non-small cell lung cancer patients (59). Taken together, these findings highlight the existence of a network of numerous CAF and MSC subpopulations in solid tumors and underline their relevance in various cancer types and across species.

## Niche Cytokines and Chemokines

The BM is a major location where hematological malignancies affecting myeloid or lymphoid lineages develop and is also an important site of metastasis for solid tumors, especially breast, prostate, and lung cancers (61, 62). The engraftment of metastatic cells from solid tumors into the BM can generate secondary tumors with either osteoblastic properties, notably in early stages of metastasis of prostate cancer, or osteolytic properties in the case of breast cancer (63–65). Osteoblastic lesions correspond to an increased bone mass at the lesion site whereas osteolytic lesions lead to a destruction of the bone structure. In the case of prostate cancer, there is a preferential accumulation of cancer cells in the lateral rather than medial endocortical bone region. This former area is enriched in osteoblasts, which could explain this phenomenon (66). The segregation of bone metastasis location between prostate and breast cancers has been extensively studied. Although this differential tropism is still not completely understood, there is a consensus about the involvement of chemokines in this phenomenon. The activity of chemokines depends on their receptors, a family of G protein-coupled seven-transmembrane-spanning molecules. Chemokines are versatile secreted factors critically required to drive the migration of immune and non-immune cells, notably within lymphoid organs including the BM. Depending on the targeted cell type, they can foster an effective anti-tumor immune response or conversely contribute to a pro-tumorigenic microenvironment. Early work from Zlotnik’s lab has shown that high production of the chemokine CXCL12 (SDF-1) by the BM is sufficient to attract breast cancer cells expressing CXCR4, one of the cognate receptors of CXCL12 (67) (**Figure 1**). CXCR4 and CXCL12 are also critical for the homeostasis of the BM ecosystem, with a key role in controlling the production and mobilization of hematopoietic stem/progenitor cells (HSPCs) (68, 69). Indeed, in the BM, HSPC niches are thought to be composed of perivascular stromal units associated with sinusoids and arterioles as reviewed recently (70). In particular, many studies have shown that a population of MSCs termed CXCL12-abundant reticular (CAR) cells overlaps with leptin receptor (LepR)-expressing cells. These CAR cells constitute a major component of HSPC niches by their capacities to produce such niche factors as CXCL12, SCF and IL-7. Similar stromal cells with salient features of CAR cells have been identified in human adult BM (10, 71). In line with these findings, the CXCL12/CXCR4 axis is key in immunosuppression and metastatic spread in solid tumors, through reciprocal crosstalk between FAP^High^ CAFs and regulatory T cells (Tregs), as well as FAP^High^ CAFs and cancer cells, respectively (24, 25, 27). In addition to CXCR4 and CXCL12, numerous studies have shown that chemokines act at different levels in the progression of the primary tumor, modulating both tumor cell proliferation, apoptosis, invasion, angiogenesis, recruitment of immune cells and resistance to chemotherapy (72–76). It appears clearly that some kind of “chemokine storm” and sustained inflammation take place in the primary tumor. This comforts the notion of a complex interplay between cancer cells, cells from the tumor microenvironment (including CAFs, MSCs and ECs) and a variety of immune cells, such as macrophages, B- and T- lymphocytes, NK cells, neutrophils and dendritic cells (73, 75, 77). The final outcome of the tumor with either sustained resistance of the host or immune escape of the tumor will depend on these interactions.

Within the tumor microenvironment, MSCs are interesting for multiple reasons. First, as stated above, these cells are highly present in the BM but can also be found at lower levels elsewhere such as in adipose tissue, lung or umbilical cord blood. They are moreover detected in multiple types of primary solid tumors (e.g., breast, ovarian, pancreatic cancers) (78–81). Several studies have shown that BM or adipose MSCs [called adipose-derived stromal cells (ADSCs)] have a particular tropism for primary tumors (81, 82). MSCs can either favor or inhibit primary tumor growth and metastasis (78, 81, 83–85)s. Recent evidence has also shown that the nature of tumor cells, in particular their low or high aggressiveness, dictates the type of interactions with MSCs and notably the production of multiple chemokines and prostaglandin E2 upon release of IL-1β by tumor cells. In turn, chemokines produced by MSCs can stimulate the invasiveness and potentially metastatic ability of tumor cells (81, 86, 87). Finally, with BM metastasis of solid tumors, interactions become possible with the other niches of BM MSCs. This interaction might favor a release of new MSCs from the BM to colonize primary tumors but may also affect the properties of MSCs themselves, notably by turning them into CAFs (81).

As stated above, growing tumors establish a chronic state of inflammation that acts locally but also systemically. The BM responds to these stress signals by remodeling the stromal landscape and expanding myeloid cells endowed with anti-inflammatory/immunosuppressive functions, further sustaining tumor progression. Several studies have reported that distant solid or diffuse tumors interfere with hematopoiesis and immune regulation within the BM. Primary breast tumors have thus been shown to generate systemic signals that mobilize BM-derived cells promoting tumor growth and dissemination. Tumor-derived factors also interfere with BM myelopoiesis, increasing the generation of granulocytic-MDSC (88, 89). In a spontaneous model of mammary carcinogenesis, Colombo’s lab revealed modifications of the representation of CXCL12-expressing BM-derived MSCs and CXCR4-expressing myeloid cells (90). Such changes in the hematopoietic compartment occurred as early as at preinvasive disease stages and were concomitant with a deregulation of circulating miRNAs. In addition, extracellular vesicles (EVs) produced by follicular lymphoma B-cells have been shown to promote the polarization of BM-derived MSCs to secrete such factors as CXCL12 that could constitute in turn a BM follicular lymphoma permissive stromal niche (91). In AML xenografts, blast-derived EVs convey endoplasmic reticulum stress *in vivo* to the animal’s BM stroma. This drives a subsequent osteo-differentiation of MSCs through the incorporation and cell-cell transfer of BMP2 by AML-derived EVs, promoting BM niche remodeling (92). Conversely, many studies provide compelling evidence that the BM can sense distant tissue transformation at premalignant/preinvasive stages and influence cancer progression. Bone-making osteoblasts have the capacity to impact distant cancer progression outside the skeleton in such tumors as melanoma, lung, and breast carcinomas. CXCL12 might constitute one of the systemic bone-derived factors that would directly promote breast cancer cell proliferation and metastasis (93). Other studies indicate that cells of the osteoblast-lineage control cancer progression in the same tumor types at least in part by mobilizing tumor-promoting myeloid cells (94, 95). Finally, BM remodeling could be beneficial or detrimental, depending on the nature of targeted hematopoietic cells, i.e., healthy vs. malignant. For instance, Belkaid’s lab recently showed that dietary restriction promoted memory T-cell accumulation in the BM. This was coordinated by glucocorticoids and associated with BM remodeling that involved an increase in such niche factors as CXCL12, erythropoiesis and adipogenesis. Consequently, this was associated with enhanced protection against infections and tumors (96). This work suggests a strategy to optimize immunological memory during nutritional challenges involving a spatiotemporal reorganization of the BM. Unfortunately, the safe haven of the BM can also be remodeled by malignant cells to disturb normal hematopoiesis. For instance, AML can shape the BM landscape to support malignant growth at the expense of normal hematopoiesis. Indeed, AML onset impaired osteogenesis as well as the production of such hematopoietic factors as CXCL12 (7). Likewise, altered cytokine expression such as a decrease of CXCL12 production in the BM of a mouse model of chronic myelogenous leukemia (CML) conferred a growth advantage to leukemia stem cells (LSCs) over normal stem cells (97). Finally, *CXCL12* deletion from MSCs reduced normal HSC numbers but promoted LSC expansion and their elimination by tyrosine kinase inhibitor treatment (98). These findings are consistent with cancer cells impairing normal hematopoiesis and provide a foundation for developing stromal-based therapies.

The relevance of stromal-based therapies is also supported in hematological malignancies by data from Hasselbalch *et al.* suggesting that chronic inflammation can be a driver of clonal evolution in patients with myeloproliferative neoplasms (MPNs) (99). In primary myelofibrosis (PMF), disease severity and treatment complexity have mainly been attributed to the association of clonal myeloproliferation and profound changes in the BM stroma, associated with an excessive production of cytokines, chemokines, growth factors and ECM components. It was initially reported that stromal changes were reactional and secondary to growth factor production by clonal hematopoietic cells. However, the presence of molecular alterations of PMF MSCs has been shown to provide an “intrinsic” osteogenic signature and an increased differentiation into osteoblasts partly dependent on endogenous TGFβ1 production and activation (100, 101). It has been suggested that the BM stroma of PMF patients is progressively inflammatory-driven by clonal hematopoietic cells towards an “autonomous” state where it becomes independent of hematopoietic cell stimulation. This in turn causes an alteration of the hematopoietic niche and participates in the amplification of the hematopoietic clone. The resulting inflammatory vicious circle becomes unbreakable in the absence of combined stroma targeted therapies (100, 102). Therefore, Stephen Paget’s theory (103) of the “seed (cancer/leukemic cell) and soil (microenvironment)” is fully sustained. However, in PMF, the bad soil (altered MSCs) endorse the bad seed (clonal HSCs), revisiting Paget’s theory in the “bad seed in bad soil” concept (104, 105). This strengthens the importance of stromal cells and their reciprocal interactions with clonal hematopoietic cells in the development and treatment of neoplasia (106).

## Cell Metabolism and Communication

While it has long been known that oxygen plays a key role in the proper functioning of mammalian cells, the mechanisms by which these cells adapt to the amount of oxygen available have only became to be understood since the 1990’s, thanks to the work of the three 2019 Nobel Prize winners in Physiology or Medicine Drs Gregg Semenza, William Kealin and Sir Peter J. Ratcliffe (107–111). The notion of hypoxia in hematopoietic niches is even more recent. While the oxygen gradient created by vascularization is understandable in solid tumors, the idea of such a gradient took longer to emerge in the world of hematopoiesis. It is now accepted, but not necessarily integrated, that the oxygen (O_2_) concentration in the hematopoietic niche varies between 1 to 4% of oxygen, strikingly different from the peripheral blood concentration of 10 to 13% (112–114). As in solid tumors, the overexpression of hypoxia-inducible factors (HIFs) has been reported in leukemia to be a marker of poor prognosis. The metabolic adaptation of tumor cells is one of the hallmarks driving aggressiveness in cancer that is clearly emphasized by low oxygen concentrations. Solid tumor cells are often glucose-addicted as sugar provides metabolic intermediates that support proliferation and migration. Thus, lactate metabolism and acidosis, other characteristics of the hypoxic tumor area, must be highly hypoxically controlled to avoid cell death (115). Based on the work of Nobel Prize winner Otto Warburg in 1931 (116), it has been speculated for decades that mitochondria were failing during the tumor process. This theory finally materialized as the Warburg effect whereby anaerobic fermentation is preferred by some tumor cells. However, several studies have now proven that mitochondria function normally in cancer cells and that blocking oxidative phosphorylation (OXPHOS) is an adaptive event (117, 118).

Although glycolytic metabolic reprogramming is common in cancer cells, several types of cells have been reported to prefer OXPHOS for energy production (119–124). AML cells thus highly depend on OXPHOS to satisfy their heightened demands for energy. Mitochondrial and OXPHOS activities greatly influence the sensitivity and *in vivo* efficacy of chemotherapeutic agents (125). Increasing evidence reveals that stromal cells affect the characteristics of cancer cells in the tumor microenvironment (126–129). The niche plays an important role in cancer cell metabolism by secreting metabolites that are used for the tricarboxylic acid (TCA)/Krebs cycle (130). Moreover, CAFs enhance the Warburg effect by interacting with cancer cells and producing lactate used by cancer cells as a fuel for mitochondrial OXPHOS (**Figure 1**). This concept is widely known as the reverse Warburg effect (131–133). Thus, the increase in reactive oxygen species (ROS) promotes the activation of HIF-1α, inducing autophagy, lysosomal degradation and loss of stromal Cav-1, consequently contributing to glycolysis in CAFs. Besides, it has been recently reported that interactions between MSCs and leukemic cells increase oxidative stress in MSCs (134) with a concomitant activation of glutathione (GSH)-based antioxidant defenses, notably through overexpression of *GPX3*, a key determinant of leukemic cell self-renewal (135, 136). These interactions also enhance leukemic blast bioenergetics by increasing OXPHOS and the TCA cycle (136). All these elements suggest that metabolic interactions within their niche are important for the maintenance of mitochondrial OXPHOS in cancer cells.

Mitochondria are not only involved in energy production through the generation of ATP by OXPHOS. They also support important anabolic reactions and are crucial regulators of apoptosis *via* the expression of molecules of the BCL-2 family at their surface (137). Horizontal transfer between two cells of mitochondria and/or mitochondrial DNA (mtDNA) *via* nanotubes, EVs or freely, is likely to have fundamental consequences for the host (**Figure 1**). A first study showed that active mitochondria and/or mtDNA from human bone marrow MSCs could rescue respiration-deficient (ρ0) lung carcinoma cells (138) and apoptotic PC12 cells (139). This effect was described in several non-cancer situations where stressed cells, frequently experiencing hypoxic or ischemic conditions, could recover after the acquisition of mitochondria from their cellular environment (reviewed in (140)). For instance, BM-derived MSCs have been shown to protect lung epithelial cells from lipopolysaccharide-induced injuries through the donation of mitochondria (141). For cancer cells, two seminal publications have shown that ρ0 cancer cells have an impaired tumorigenic potential that can be restored, together with respiration, by the transfer of mtDNA (142) or active mitochondria (143) from surrounding cells, both *in vitro* and *in vivo*. Interestingly, it has also been demonstrated that CAF-derived EVs can transfer mtDNA to OXPHOS-deficient breast cancer cells, leading to the restoration of mitochondrial metabolic activities (144). MSCs moreover could transfer active mitochondria to AML leukemic blasts, especially upon sensitization of leukemic cells by chemotherapy, probably, among other still unclear mechanisms, *via* AML cell-derived ROS (145, 146). It was also demonstrated that MSCs recognize damaged mitochondria released by leukemic cells under chemotherapy as danger signals and react by stimulating mitochondrial biogenesis followed by transfer of active mitochondria to AML cells (147). Another interesting study showed that BM MSCs from acute lymphoblastic leukemia (ALL) patients harbor a CAF phenotype. Upon chemotherapy and ROS induction, they transfer mitochondria to ALL blasts to support their survival and resistance to chemotherapy (148). The uptake of mitochondria by leukemic cells can increase their mitochondrial mass by up to 14% (145) and is associated with better fitness and a higher resistance to chemotherapy. Since mitochondria-recipient cells become able to resist apoptotic signals, it is possible that this transfer could increase the pool of anti-apoptotic molecules of the BCL-2 family in leukemic blasts. Another obvious effect of mitochondrial transfer is an increase in ATP content (145, 147, 149) and in other important metabolites. A recent study demonstrated that transferred mitochondria were important to sustain pyrimidine synthesis and cell proliferation *via* the dihydroorotate dehydrogenase (DHODH) enzyme present in the mitochondrial membrane (150). Exogenous mitochondria could also support resistance to ferroptosis cell death as DHODH appears to mediate an important protective pathway against ROS-induced lipid peroxidation that triggers ferroptosis (151). Finally, mitochondrial transfer could modulate immune responses as it has been reported that horizontal transfer from MSCs could trigger Treg differentiation to limit tissue damage and inflammation during graft-*versus*-host disease (152). Whether this phenomenon also occurs in the BM hematopoietic niche and affects other lymphoid cell subsets such as cytotoxic T-lymphocytes or NK cells during leukemia development remains to be studied.

## Vascular Niche, Angiogenesis and Endothelial Plasticity

The vascular endothelium refuels the tumor mass with oxygen and metabolites and settles a favorable microenvironment for tumor growth. This is strikingly illustrated in tumors from the central nervous system, where homeostasis of the cerebral vasculature is crucial. As for embryonic and adult stem cells, cancer stem cells reside within a niche articulated around vascular units (153), defined as the vascular niche. This environment allows privileged control of metabolic conditions, secreted protein dosage as well as fine-tuned regulation of cell adhesion and communication with the surrounding ECM and neighboring ECs (154). Cancer stem cells are indeed located in the close vicinity of tumor blood vessels where ECs are suspected to dictate stem cell identity (155, 156). The concept of (peri)vascular niche is also highly significant in the BM and has evolved through the better characterization of HSPCs. In mice, the HSPC compartment is functionally and molecularly heterogeneous, due in part to an extrinsic control by the BM microenvironment, including ECs. Indeed, recent advances in cell imaging and HSPC reporter-mice have revealed the association of HSPCs with at least two types of blood vessels. The latter are central endothelium featuring sinusoids (157) and an endosteal arterial/arteriolar endothelium which is close to bone diaphysis and epiphysis and defines transition vessels (158) (**Figure 1**). Sinusoidal and endosteal ECs differ phenotypically, the latter expressing high levels of endomucin and CD31, while sinusoidal ECs display low levels of both these markers. The location of endosteal ECs in bone metaphyses, close to osteoprogenitor cells, allows for an efficient coupling between osteogenesis and angiogenesis. Furthermore, sinusoidal and endosteal ECs are surrounded by unique specific perivascular MSCs. Although most CXCL12 and SCF in the BM is produced by CAR/LepR+ cells (159, 160), ECs are also a source of both niche factors and hence are involved in the hematopoiesis process. Arterial and transition vessel ECs by displaying such a higher expression of CXCL12 and SCF maintain HSPC quiescence, while sinusoidal vessels, fenestrated and more permeable, promote BM cell trafficking (158, 161, 162).

Several studies have revealed the role of the BM vasculature in the development of leukemia and chemoresistance. In AML, vascular niches provide signals that regulate proliferation and stem cell-like properties (163, 164). In a reciprocal way, AML cells release inflammatory cytokines that activate the vascular endothelium, inducing the expression of such adhesion molecules as VCAM1, promoting AML proliferation and chemoresistance (165, 166). External cues emanating from ECs can regulate the fate of cancer stem cells both in solid tumors and leukemia. In cerebral tumors, exploration of the endothelial secretome identified the vasopeptide apelin (APLN) as a central regulator for endothelial-mediated maintenance of patient-derived glioma stem-like cells *in vitro* and *in vivo* (167). Further studies confirmed the instrumental role of APLN to sustain tumor cell expansion and progression (168). Likewise, a subpopulation of APLN-expressing ECs in the BM orchestrates HSPC maintenance, and further repopulation in the therapy-induced damaged bone microenvironment (169). In luminal breast carcinoma, the BMP2 was found to be overproduced by ECs from the tumor stroma. This factor is an important actor of the stem cell niche, participating also in the initiation of stem cell transformation (170).

In hematological malignancies, sinusoidal ECs from the BM vascular niche of patients with chronic myeloid leukemia have been shown to be the main source of BMP2 and BMP4, involved in the maintenance and expansion of leukemic stem cells (171). BMP4 overproduction in the AML microenvironment furthermore contributes to blast cells “reprogramming” towards a stem-cell like phenotype (172). In addition, BMP4 produced by the leukemic microenvironment is involved in leukemic stem cell quiescence mediated by Jak2/Stat3 signaling and contributes to relapse and tumor escape (173) (**Figure 1**). Similar data in solid tumors, from many laboratories, have identified the BMP-signaling pathway as a major driver of BM dormancy (174).

Seed and soil interactions have to be considered as reciprocal, signals provided by cancer cells impacting ECs and *vice versa*. The influence of cancer cells towards EC is to promote angiogenesis and increase vascular permeability to respectively provide the oxygen required for growth and allow for cell dissemination. In solid tumors, pro-angiogenic factors (VEGF, Sema3A), either soluble or delivered through tumor-derived EVs, contribute to an increase of both angiogenic potential and permeability (175, 176). Malignant hematopoietic cells are high consumers of oxygen and evolve in a hypoxic environment that favors angiogenesis. An increase in BM vascular density and angiogenic markers (VEGF-A, FGF2, VEGF-R) has been highlighted in several hematological malignancies (177, 178). The sites of active angiogenesis in tumor BM niches are still not fully characterized, but ECs in the transient zone close to the endosteal niche could mediate the local growth of blood vessels in normal bone (158). In MPNs and leukemia, neo-vessels are also characterized by an abnormal tortuous architecture (178, 179). In MPNs, increased microvascular density and expression of VEGF have been reported to correlate with the allelic charge of *JAK2*-V617F mutation (180, 181). In a subset of thrombotic MPN patients, this mutation has been detected in hepatic and splenic ECs as well as in endothelial progenitors, suggesting their clonality (182). More recently, introduction of the *JAK2*-V617F mutation in ECs has been shown to modify these cells towards a pro-adherent and pro-thrombotic profile (183, 184). These results suggest that, similarly to MSCs, ECs may have acquired intrinsic modifications that participate in the activated/inflammatory state within the BM niche and in leukemic progression. An aberrant increase in permeability is an additional striking feature of tumor blood vessels (185) which strongly alters drug delivery in solid tumors (186). An increased permeability of BM vessels, induced by leukemic cells, could also be associated with an impaired perfusion hampering normal hematopoiesis and supporting malignancy as shown in an AML patient-derived xenograft model (166).

Beside their role in angiogenesis, ECs may also engage in the dynamic process of endothelial-to-mesenchymal transition (EndMT), which drives reprogramming of ECs towards a mesenchymal phenotype (187). Initially described in normal cardiac development, this plasticity has been highlighted in several solid tumors in response to tumor environmental soluble and/or mechanical cues, as well as upon therapeutic assaults (188). EndMT may provide a source of CAFs (189) and contribute to metastasis dissemination by destabilizing the endothelial barrier (190). Furthermore, EndMT has been described as a tumor arm to resist chemo- and radio-therapies (191, 192). Recent data support such a transition process in regenerative human BM, as a subset of ECs in trabecular sinusoid vessels has been shown to display an EndMT transcriptional signature (193). Importantly, this endothelial derived-mesenchymal population harbors properties of pluripotent stromal cells, with multi-lineage differentiation capacity (adipocyte, osteoblast, chondrocyte) and supportive capacity of hematopoiesis. Whether EndMT plays a role in hematological cancer is not confirmed yet, but this process surely could participate in the reconstitution of the hematopoietic BM niche after therapy (193). In the BM and spleen of PMF patients, the presence of microvascular ECs showing functional and morphologic changes associated with the MSC phenotype is in agreement with the potential contribution of EndMT to the BM fibrosis process that characterizes this disease (194).

## Conclusion

This review highlights how knowledge is progressing, in both solid tumors and hematological malignancies, in identifying the role of the multiple subsets of cells widely referred to as “cells of the microenvironment”. The latter clearly constitute a network of interacting subsets, which are increasingly well identified, but still incompletely understood. From cytokine/chemokine release patterns to interactions with angiogenesis and oxygen regulation, much remains to be deciphered. However, this review clearly highlights that solid tumors and hematological malignancies use similar strategies to survive in a microenvironment dedicated to their suppression, in particular by modifying the microenvironment to adapt it to tumor growth, while altering its physiological role.

Information generated by single-cell analyses can be used as a blueprint for the identification of CAFs or MSCs subtypes in various organs in different pathological conditions. Comparison of CAF subtypes’ molecular profiles with those of MSCs will be useful to identify potential mechanistic similarities in tumor inflammation and niche alterations across malignancies. Much remains to be done however before transposing the results obtained in mouse models to the primary cells of human tumors and hematologic malignancies.

It is obviously still needed to discover specific means to interfere with the intricate interplay between niche actors that affect cancer/leukemic growth and prevent leukemia relapse. The power of multi-omic analyses of the tumor microenvironment, associated with a pan-tumor integrative approach of cancer niche abnormalities could be decisive in proposing new therapeutic strategies targeting niches in order to eradicate cancer cells.

## Author Contributions

All authors made extensive reviews of the literature listed and drafted different sections of the review. NMM drew the figure, which was finalized with the help of SM and OH. OH conceived, designed, supervised and finalized the review. All authors contributed to the article and approved the submitted version.

## Funding

This study was supported by the GDR 3697 Micronit “Microenvironment of Tumor niches”, a CNRS research network which has been running in France for seven years.

## Conflict of Interest

The authors declare that the research was conducted in the absence of any commercial or financial relationships that could be construed as a potential conflict of interest.

## Publisher’s Note

All claims expressed in this article are solely those of the authors and do not necessarily represent those of their affiliated organizations, or those of the publisher, the editors and the reviewers. Any product that may be evaluated in this article, or claim that may be made by its manufacturer, is not guaranteed or endorsed by the publisher.
